# The Impact of Multimorbidity Burden, Frailty Risk Scoring, and 3-Directional Morphological Indices vs. Testing for CSF Responsiveness in Normal Pressure Hydrocephalus

**DOI:** 10.3389/fnins.2021.751145

**Published:** 2021-11-16

**Authors:** Shereen X. Y. Soon, A. Aravin Kumar, Audrey J. L. Tan, Yu Tung Lo, Christine Lock, Sumeet Kumar, Janell Kwok, Nicole C. Keong

**Affiliations:** ^1^Department of Neurosurgery, National Neuroscience Institute, Singapore, Singapore; ^2^Department of Neuroradiology, National Neuroscience Institute, Singapore, Singapore; ^3^Duke-NUS Medical School, Singapore, Singapore

**Keywords:** normal pressure hydrocephalus, external lumbar drainage, ventricular morphology, ventricular volume, ADNI database

## Abstract

**Objective:** Multimorbidity burden across disease cohorts and variations in clinico-radiographic presentations within normal pressure hydrocephalus (NPH) confound its diagnosis, and the assessment of its amenability to interventions. We hypothesized that novel imaging techniques such as 3-directional linear morphological indices could help in distinguishing between hydrocephalus vs. non-hydrocephalus and correlate with responsiveness to external lumbar drainage (CSF responsiveness) within NPH subtypes.

**Methodology:** Twenty-one participants with NPH were recruited and age-matched to 21 patients with Alzheimer’s Disease (AD) and 21 healthy controls (HC) selected from the Alzheimer’s Disease Neuroimaging Initiative (ADNI) database. Patients with NPH underwent testing *via* the NPH programme with external lumbar drainage (ELD); pre- and post-ELD MRI scans were obtained. The modified Frailty Index (mFI-11) was used to stratify the NPH cohort, including Classic and Complex subtypes, by their comorbidity and frailty risks. The quantitative imaging network tool 3D Slicer was used to derive traditional 2-dimensional (2d) linear measures; Evans Index (EI), Bicaudate Index (BCI) and Callosal Angle (CA), along with novel 3-directional (3d) linear measures; z-Evans Index and Brain per Ventricle Ratio (BVR). 3-Dimensional (3D) ventricular volumetry was performed as an independent correlate of ventriculomegaly to CSF responsiveness.

**Results:** Mean age for study participants was 71.14 ± 6.3 years (18, 85.7% males). The majority (15/21, 71.4%) of participants with NPH comprised the Complex subtype (overlay from vascular risk burden and AD); 12/21 (57.1%) were Non-Responders to ELD. Frailty alone was insufficient in distinguishing between NPH subtypes. By contrast, 3d linear measures distinguished NPH from both AD and HC cohorts, but also correlated to CSF responsiveness. The z-Evans Index was the most sensitive volumetric measure of CSF responsiveness (*p* = 0.012). Changes in 3d morphological indices across timepoints distinguished between Responders vs. Non-Responders to lumbar testing. There was a significant reduction of indices, only in Non-Responders and across multiple measures (z-Evans Index; *p* = 0.001, BVR at PC; *p* = 0.024). This was due to a significant decrease in ventricular measurement (*p* = 0.005) that correlated to independent 3D volumetry (*p* = 0.008).

**Conclusion.** In the context of multimorbidity burden, frailty risks and overlay from neurodegenerative disease, 3d morphological indices demonstrated utility in distinguishing hydrocephalus vs. non-hydrocephalus and degree of CSF responsiveness. Further work may support the characterization of patients with Complex NPH who would best benefit from the risks of interventions.

## Introduction

The diagnosis of normal pressure hydrocephalus (NPH), first termed by Hakim and Adams (1965), requires supportive evidence from clinical history, physical examination and brain imaging (Schirinzi et al., 2015). It is characterized by the clinical triad of gait disturbance, mental deterioration and urinary incontinence, along with the enlargement of the cerebral ventricles (Relkin et al., 2005). Although the precise global incidence and prevalence of NPH are not known, NPH has been found to mainly implicate the geriatric population (Tanaka et al., 2009; Jaraj et al., 2014). Patients with NPH are thus also known to present with burden of concurrent comorbidities. Although attempts have been made to provide supplementary guidance for the needs of this challenging population (Malm et al., 2013), this cohort has previously been excluded from standard practice guidelines (Bergsneider et al., 2005; Klinge et al., 2005; Marmarou et al., 2005b, c; Relkin et al., 2005). We have previously described a particular subtype of NPH, with a heavy burden of concurrent comorbidities, termed “Complex NPH” as per Lock et al. (2019). Here, we have expanded on our definition for consistency, and further refined it *via* this work, as a subtype of NPH patients matching the following criteria:–(i) clinical symptoms and signs consistent with probable/possible NPH according to international/Japanese guidelines, (ii) with strong neuroradiological features supportive of the NPH diagnosis (such as DESH or other imaging biomarkers), (iii) but presenting with overlay from multiple comorbidities co-existing (such as significant cardiovascular risk burden or neurodegenerative disorders), and (iv) who are difficult to test using standard supplementary measures (due to poor cognitive/gait/balance/functional ability) or high risk for testing/surgical interventions (due to cardiac disease, antiplatelet or anticoagulation therapy, or spinal operations). In this cohort, invasive gold-standard testing may be difficult or inconclusive; the needs of such patients have elevated the importance of developing more precise risk stratification scoring and neuroimaging tools to characterize responsiveness to CSF drainage.

Given early and accurate diagnosis, symptoms of Classic NPH (gait disturbance, dementia, incontinence) can be reversed through ventricular shunting (Shprecher et al., 2008). There is also evidence that, in patients with Complex NPH, there is still a remediable component of responsiveness to external lumbar drainage (CSF responsiveness) that is amenable to interventions. External lumbar drainage (ELD), involving the drainage of cerebrospinal fluid (CSF) through a lumbar spinal catheter over several days, is a gold-standard supplementary test for shunt responsiveness (Ishikawa et al., 2008; Gallina et al., 2018). Several guidelines for the management of NPH have reported high sensitivities (50–100%) and high positive predictive values (80–100%) for the prognostic value of ELD (Walchenbach et al., 2002; Marmarou et al., 2005a; Ishikawa et al., 2008; Chotai et al., 2014). However, the perioperative morbidity of CSF shunting procedures are also significant (38% pooled rate of shunt complication including death, infection, seizures, shunt malfunction, subdural hemorrhage or effusion) (Hebb and Cusimano, 2001). Furthermore, improvements in cognitive deterioration have been found to be limited to only 30–50% of shunted patients (Black, 1980; Vanneste, 2000). Thus, there is a need for non-invasive supplementary screening tools to aid in the diagnostic and prognostic selection for shunt-responsive NPH patients.

By current clinical standards, 2-dimensional (2d) morphological indices such as the Evans Index (EI), Bicaudate Index (BCI) and Callosal Angle (CA), are used as diagnostic markers in differentiating NPH cohorts to AD and healthy control (HC) cohorts (Marmarou et al., 2005a; Ishikawa et al., 2008). However, recent volumetric studies demonstrating z-axial, as opposed to x-axial, ventricular expansion have suggested 2d morphological indices may be insufficient to fully describe the patterns of ventricular enlargement vs. brain atrophy across NPH and AD cohorts (Ambarki et al., 2010; Toma et al., 2011). Traditional linear morphological indices have also been found to be inadequate in characterizing intra-NPH cohorts such as NPH and secondary NPH due to variations in fluid distribution patterns within NPH cohorts (Yamada et al., 2017).

Other studies involving the use of morphological indices in NPH cohorts have since supported the potential utility of novel 3-directional (3d) linear indices (z-Evans index and brain per ventricle ratio, BVR) toward the differential diagnosis of NPH and AD cohorts (Yamada et al., 2016, 2017). The attractiveness of such methods are that 3d linear indices not only describe the directional expansion of brain ventricles, but are able to also characterize differences in fluid distribution patterns across differing CSF compartments amongst these disease cohorts.

In this study, we examined the use of a 3d morphological methodology to distinguish between cohorts with hydrocephalus vs. non-hydrocephalus and evaluated its performance in Asian patients with NPH, across both Classic and Complex subtypes (Lock et al., 2019). Here we present our findings on the relevance of multimorbidity burden and frailty risks and their associations between 3d linear indices, changes in ventricular size and responsiveness to CSF responsiveness *via* ELD.

## Materials and Methods

### Data Source

Twenty-four patients with probable NPH (mean age 71 ± 6.3 years) who underwent the extended CSF drainage protocol *via* the NPH programme at the National Neuroscience Institute, Singapore, were recruited prospectively. All patients met criteria for probable or possible NPH according to published guidelines (Relkin et al., 2005), presenting with ventriculomegaly and at least one of three features of the NPH clinical triad. Additional details of the protocol have been previously published (Lock et al., 2019). Participants had one pre-intervention baseline MR scan and one post-intervention MR scan after CSF lumbar drainage (≥300 ml of CSF drained over 3 days, or otherwise determined by the treating consultant). Participants either had a lumbar drain insertion, or serial taps from an Ommaya reservoir. Three participants were excluded from analysis—one was unable to undergo MR scanning, another participant did not complete CSF drainage and was discontinued from the study due to a subarachnoid hemorrhage, and the third participant did not proceed with CSF drainage due to an incidental finding during pre-intervention clinical investigation. The study was approved by the National Healthcare Group Domain Specific Review Board (NHG DSRB; Ref 2014/00838) and the SingHealth Centralised Institutional Review Board (CIRB; Ref 2016/2627). Informed consent was obtained from all participants or their legal representatives, if applicable.

Data for comparator groups of twenty-one age-matched AD patients (9 males, mean age 73 ± 8.6 years) and healthy controls (HC; 7 males, mean age 73 ± 3.4 years) were obtained from baseline scans of patients enrolled to the ADNI 1 phase of the Alzheimer’s Disease Neuroimaging Initiative (ADNI) study^1^. AD patients had mild AD, meeting NINCDS-ADRDA criteria for probable AD and a Clinical Dementia Rating of 0.5 or 1.0 (Alzheimer’s Disease NeuroImaging1(ADNI1)Clinical Protocol, 2005; Petersen et al., 2010).

### Image Acquisition and Pre-processing

MR imaging data for NPH participants were acquired with a 3-T MR Philips scanner (Ingenia, Philips Medical Systems, Best, the Netherlands), including 3D T1, T2, FLAIR, and DTI sequences. Three-dimensional axial T1-weighted imaging with sensitivity encoding (SENSE) was acquired (*TR* = 7.3ms, *TE* = 3.3 ms, flip angle = 8°, FOV = 256 × 256 mm, voxel size = 1.0 × 1.0 × 1.0 mm). Eight patients were downgraded to the 1.5-T scanner at equivalent specifications due to institutional MR safety protocol. AD and HC participants from ADNI were scanned in a 3-T MRI scanner (GE Healthcare, Philips Medical Systems, or Siemens Healthcare, depending on the ADNI scanning site). MRI scanning protocols for each scanner model are available online^2^.

### Modified Frailty Index-11

Frailty was quantified using the *modified Frailty Index-11* (mFI-11) and its components found in Table 1. The mFI-11 is a validated shortened version of the 70-point Canadian Study of Health and Aging Frailty Index (CSHA FI) (Fang et al., 2017). The mFI-11 is one of the more commonly utilized tool to assess frailty in various surgical subspecialities as it examines easily identifiable clinical information that can be extracted from available clinical notes, or obtained at bedside, with statistically simple and reproducible calculations (Pazniokas et al., 2020). Patients were given a binary score for each comorbidity assessed, then stratified by the extent of their frailty based on their cumulative scores (mFI-11 score: 0–2 and ≥3; with the highest mFI-11 score in our cohort being 6).

**TABLE 1:** Clinical characteristics of normal pressure hydrocephalus (NPH) cohort.

Characteristic	Number of subjects (*n = 21*)
**Gender**	
Male	18 (85.7)
Female	3 (14.3)
**Mean age, years (SD)**	71.14 (± 6.3)
**Category of normal pressure hydrocephalus (NPH)**	
Classic	6 (28.6)
Complex	15 (71.4)
**External lumbar drainage (ELD) responsiveness**	
Responder	9 (42.9)
Non-responder	12 (57.1)
**Modified Frailty Index-11 (mFI-11)**	
Hypertension	17 (81.0)
Impaired sensorium	13 (61.9)
Diabetes mellitus	7 (33.3)
Activities of daily living dependent	6 (28.6)
Myocardial infarction	3 (14.3)
Percutaneous coronary intervention/angina	3 (14.3)
Chronic/acute respiratory disease	2 (9.5)
Peripheral vascular disease	1 (4.8)
Coronary heart failure	0
Cerebrovascular accident/transient ischemic attack	0
mFI-11 score: 0–2	9 (42.9)
mFI-11 score: ≥3	12 (57.1)

*Complex NPH; term as per Lock et al. (2019) and further refined in this work, a subtype of NPH patients matching the following criteria:- (i) clinical symptoms and signs consistent with probable/possible NPH according to international and Japanese guidelines, (ii) with strong neuroradiological features supportive of the NPH diagnosis (such as DESH or other imaging biomarkers), (iii) but presenting with overlay from multiple comorbidities co-existing (such as significant cardiovascular risk burden or neurodegenerative disorders) and (iv) who are difficult to test using standard supplementary measures (due to poor cognitive/gait/balance/functional ability) or high risk for testing/surgical interventions (due to cardiac disease, antiplatelet, or anticoagulation therapy or spinal operations).*

### Morphological Features

We used the open-source quantitative imaging network tool, 3D Slicer 4.9, to derive traditional 2-dimensional (2d) linear and 3-directional (3d) linear measurements, as well as 3-Dimentional (3D) quantitative ventricular volumes^3^ (Fedorov et al., 2012). This tool was selected as it functions akin to a radiology workstation that allows for versatile visualizations, but also provides advanced modular functionalities such as semi-automated segmentations, volumetry, and 3D quantitative measurements. This combined functionality allowed us to streamline optimization steps usually performed at the scanner workstation with subsequent morphological measurements, within a single continuous workflow.

**FIGURE 1:**
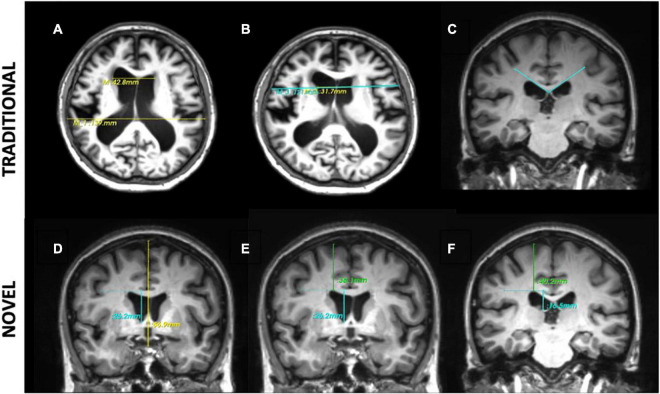
Morphological indices. Traditional 2-dimensional (2d) linear measures: **(A)** Evans Index is the ratio of the maximal width of the frontal horns of the lateral ventricles (top line) to the maximal width of the internal diameter of the cranium (bottom line). **(B)** Bicaudate Index is the ratio of the maximum intercaudate distance (yellow line) to the width of the brain along the same line on the axial plane (blue line). **(C)** The callosal angle is measured as the angle of the corpus callosum through the posterior commissure perpendicular to the ACPC plane. Novel 3-directional (3d) linear measurements: **(D)** the z-Evans index is the ratio of the maximum z-axial length of the frontal horns of the lateral ventricles (blue line) to the maximum cranial z-axial length on the coronal plane (yellow line), perpendicular to the ACPC plane, on the anterior commissure. **(E,F)** The Brain-Ventricle-Ratios (BVRs) measured at the level of the anterior and posterior commissure, respectively, are measured as the maximal brain thickness above the lateral ventricles (blue line) divided by the maximum length of the lateral ventricles (green line), on the coronal plane.

Firstly, we reproduced the methodology of deriving measurements of morphological indices inaccordance with the work by Yamada et al. (2017), which includes traditional 2d linear measurements- Evans Index (EI), Callosal Angle (CA), Bicaudate Index (BCI) and 3d linear measurements- z-Evans, Brain-Ventricle Ratio (BVR). Intraclass Correlation Coefficients (ICCs) showed good intra-rater agreement for traditional 2d linear measures (EI, ICC = 0.980; CA, ICC = 0.953) and 3d linear measures (z-Evans, ICC = 0.967; BVR at AC, ICC = 0.972, BVR at PC, ICC = 0.989). The EI (Figure 1A) was defined as the ratio of the maximal width of the frontal horns of the lateral ventricles to the maximal width of the internal diameter of the cranium. The BCI (Figure 1B) was defined as the ratio of the maximum intercaudate distance to the width of the brain along the same line on the axial plane (Chen et al., 2017). The callosal angle (Figure 1C) was defined as the angle of the roof of the bilateral ventricles on the coronal plane at the level of the posterior commissure (PC). The z-Evans index (Figure 1D) was defined as the ratio of the maximum z-axial length of the frontal horns of the lateral ventricles to the maximum cranial z-axial length on the coronal plane, perpendicular to the anterior commissure-posterior commissure (ACPC) line. The brain-ventricle ratios (BVRs) at the AC and PC (Figures 1E,F) were measured as the maximal brain width above the lateral ventricles divided by the maximum height of the lateral ventricles, on the coronal plane with reference to the AC and PC levels, respectively. 3-Dimensional (3D) volumetric measures of ventricles were also derived in this study to validate results from their linear counterparts.

**FIGURE 2:**
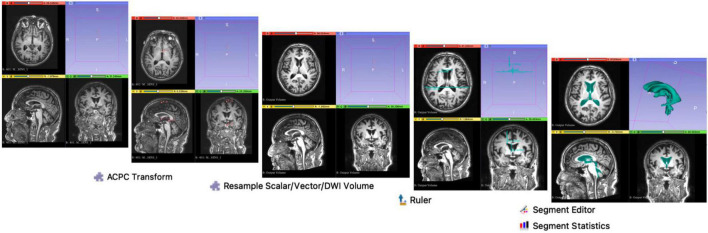
Methodology on 3D Slicer. Using the *ACPC Transform* and the *Resample Scalar/Vector/DWI Volume* modules, planes of the T1-weighted DICOM scans were re-aligned parallel to the ACPC line for accurate replication of the alignment at a typical radiology workstation, as per Yamada et al. (2017). *Ruler Module* was used to extract measurements for the various morphological indices. *Segment Editor* was used for the semi-automated segmentation of the ventricles. *Segment Statistics* was used to derive ventricular volumes by automatic counting of voxels in segments.

A flow chart of the methodology used on 3D Slicer is illustrated in Figure 2. Using the *ACPC Transform* and the *Resample Scalar/Vector/DWI Volume* modules, planes of the T1-weighted DICOM scans were re-aligned parallel to the ACPC line for accurate replication alignment at a radiology workstation. *Ruler Module* was used to extract measurements for the various morphological indices. *Segment Editor* was used for the semi-automated segmentation of the ventricles. *Segment Statistics* was used to derive ventricular volumes by semi-automatic counting of voxels in segments.

Validation of our workflow on 3D Slicer was done by comparing inter-cohort morphological trends of our NPH, AD and HC cohorts to that of Yamada et al. (2016), which was conducted on a Siemens workstation (Yamada et al., 2017; Supplementary Figure 1). The lower BVR measurements between AD cohorts could be accounted for by the differences in the AD screening criteria set by ADNI Petersen et al. (2010) and Yamada et al. (2017) resulting in a more severe AD cohort in our study.

### Statistical Analysis

Categorical data were described as numbers and percentages, continuous variables were reported as mean and standard deviation (SD). Categorical variables were compared and analyzed with Fisher’s Exact test. Inter- and intra-group comparisons for morphological measures were tested with independent-samples Mann-Whitney *U* test and Wilcoxon signed rank test. All statistical tests were two-tailed and statistical significance was assumed at p < 0.05. The statistical analyses were performed using R statistical software version 3.3.3. (R Core Team, 2021)^4^.

## Results

### Clinical Characteristics

Twenty-one patients (mean age 71 ± 6.3 years; 18 males, 3 females) with NPH were recruited. Of these 21, six were found to be solely consistent with criteria for NPH diagnosis as per international guidelines and classified as Classic NPH; fifteen patients met both the international criteria for their clinico-radiological presentation and our described definition for Complex NPH. Subjects were classified as responders to ELD if their levels of improvement met the minimal clinically important difference (MCID) in at least one domain of NPH symptomatology sufficient to support consideration for shunt insertion. We defined the MCID using the following thresholds: an increase of ≥10% in any measure of inpatient gait, balance, or cognitive testing, matched with a ≥20% functional improvement reported by the patient or caregiver at home following discharge. Using this criteria, there were 9 Responders and 12 Non-Responders to CSF drainage *via* ELD within our NPH cohort. A summary of the demographics of our cohort is found in Table 1.

### Frailty

Following frailty stratification by the mFI-11, 9/21 (42.9%) and 12/21 (57.1%) had a mFI-11 score of 0–2, and ≥3, respectively. The cohort was stratified relatively evenly with respect to ELD response *(p* = *1.00)*, and a majority of the frailer patients (mFI-11 ≥ 3) were classified as Complex NPH (Classic NPH 3/6, 50% vs. Complex NPH 9/15, 60%; *p = 1.00*). The distribution of our NPH subtypes (Responders vs. Non-Responders and Classic vs. Complex groups) stratified by frailty can be found in Table 2.

**TABLE 2:** Stratification of frailty risks of the normal pressure hydrocephalus (NPH) cohort.

mFI-11 group	Responder (*n* = 9)	Non-responder (*n* = 12)	*p*-value	Classic (*n* = 6)	Complex (*n* = 15)	*p*-value
0–2	4 (44.4)	5 (41.7)	1	3 (50)	6 (40)	1
≥3	5 (55.6)	7 (58.3)		3 (50)	9 (60)	

*mFI-11; Modified Frailty Index-11.*

*Complex NPH; term as per Lock et al. (2019) and further refined in this work, a subtype of NPH patients matching the following criteria:- (i) clinical symptoms and signs consistent with probable/possible NPH according to international and Japanese guidelines, (ii) with strong neuroradiological features supportive of the NPH diagnosis (such as DESH or other imaging biomarkers), (iii) but presenting with overlay from multiple comorbidities co-existing (such as significant cardiovascular risk burden or neurodegenerative disorders) and (iv) who are difficult to test using standard supplementary measures (due to poor cognitive/gait/balance/functional ability) or high risk for testing/surgical interventions (due to cardiac disease, antiplatelet, or anticoagulation therapy or spinal operations).*

### Morphological Indices: Inter-Cohort Comparisons

Both 2d and 3d linear measurements were able to distinguish between disease cohorts (NPH vs. AD), and between NPH and healthy controls (*p < 0.001*), as seen in Table 3. NPH patients also had significantly larger ventricles (characterized by higher EI and BCI values) and significantly tight high-convexities (characterized by lower CA and BVR values) in their morphology.

**TABLE 3:** Inter-cohort comparisons of normal pressure hydrocephalus (NPH) vs. non-NPH *via* baseline morphological indices.

Baseline measures				*p*-values		
	NPH	AD	HC	NPH–AD	NPH–HC	AD–HC
**Traditional 2d linear measures**						

EI	0.380 ± 0.053	0.286 ± 0.033	0.277 ± 0.028	<0.001*	<0.001*	0.315
BCI	0.284 ± 0.027	0.186 ± 0.032	0.175 ± 0.025	<0.001*	<0.001*	0.216
Callosal angle (degrees)	57.1 ± 20.8	109.0 ± 15.1	106.4 ± 11.5	<0.001*	<0.001*	0.528

**Novel 3-directional linear measures**						

z-Evans index	0.448 ± 0.056	0.294 ± 0.037	0.273 ± 0.043	<0.001*	<0.001*	0.096
BVR at AC	0.678 ± 0.176	1.436 ± 0.257	1.589 ± 0.332	<0.001*	<0.001*	0.104
BVR at PC	0.835 ± 0.316	2.611 ± 0.964	3.281 ± 1.220	<0.001*	< 0.001*	0.055

*Significant at α < 0.05.

*EI, Evans index.*

*BCI, Bicaudate index.*

*BVR, Brain-ventricle ratio.*

*AC, Anterior commissure.*

*PC, Posterior commissure.*

*NPH, Normal pressure hydrocephalus.*

*AD, Alzheimer’s disease.*

*HC, Healthy control.*

*NPH cohorts consisted of classic and complex NPH patients. Differences in traditional 2d linear measures (EI, BCI, Callosal Angle) and 3-directional linear measures (z-Evans Index, BVR at AC and PC level), between NPH and AD, and between NPH and HC, were significant. No significant differences observed between AD and HC cohorts.*

### Morphological Indices: Intra-Cohort Comparisons

Compared to our patients with Complex NPH, our patients with the classic NPH subtype demonstrated a trend toward relatively larger ventricles (higher EI, BCI, and z-Evans index values) and tighter high-convexities (higher CA and lower BVR values) (Table 4). However, neither traditional 2d nor novel 3d linear measures distinguished between the two subtypes.

**TABLE 4:** Intra-cohort comparisons of Classic vs. Complex normal pressure hydrocephalus (NPH) *via* baseline morphological indices.

Pre-ELD measurements	Classic NPH (*n* = 6)	Complex NPH (*n* = 15)	*p*-value
**Traditional 2d linear measures**			

EI	0.392 ± 0.0577	0.373 ± 0.0534	0.494
BCI	0.287 ± 0.0261	0.281 ± 0.0279	0.779
Callosal Angle (degrees)	67.1 ± 23.9	54.6 ± 18.6	0.248

**Novel 3-directional linear measures**			

z-Evans index	0.436 ± 0.0588	0.449 ± 0.0565	0.602
BVR at AC	0.712 ± 0.158	0.674 ± 0.188	0.547
BVR at PC	0.922 ± 0.356	0.814 ± 0.312	0.494

*ELD, External lumbar drain.*

*EI, Evans index.*

*BCI, Bicaudate index.*

*BVR, Brain-ventricle ratio.*

*AC, Anterior commissure.*

*PC, Posterior commissure.*

*Comparison of pre-ELD morphological indices between NPH subtypes (Classic vs. Complex). No significant differences were observed between Classic and Complex NPH cohorts.*

As there were no significant differences in morphological indices found between Classic vs. Complex NPH pre-testing, for subsequent comparisons between cohorts performed pre- and post-ELD, we considered these subtypes as one NPH cohort. Differences in morphological indices revealed a significant decrease in the z-Evans index values post-ELD (*p* = 0.012), and non-significant decreases in the EI and BCI values (Table 5).

**TABLE 5:** Comparison of normal pressure hydrocephalus (NPH) cohorts pre- and post-lumbar testing using morphological measures, regardless of responsiveness to external lumbar drainage (ELD).

Measurements	Pre-ELD	Post-ELD	*p*-value
**Traditional 2d linear measures**			

EI	0.380 ± 0.053	0.377 ± 0.051	0.437
BCI	0.284 ± 0.027	0.282 ± 0.031	0.662
Callosal angle (degrees)	57.1 ± 20.8	58.1 ± 20.2	0.344

**Novel 3-directional linear measures**			

z-Evans index	0.448 ± 0.056	0.440 ± 0.055	0.012*
BVR at AC	0.678 ± 0.176	0.672 ± 0.196	0.792
BVR at PC	0.835 ± 0.316	0.860 ± 0.284	0.233

*Significant at α < 0.05.

*ELD, External lumbar drain.*

*EI, Evans index.*

*BCI, Bicaudate index.*

*BVR, Brain-ventricle ratio.*

*AC, Anterior commissure.*

*PC, Posterior commissure.*

*Comparison of pre- and post-ELD morphological indices within NPH cohort. As there were no significant differences in morphological indices found between Classic vs. Complex NPH pre-testing, for subsequent comparisons between cohorts performed pre- and post-ELD, we considered these subtypes as one NPH cohort. Differences in traditional 2d linear measures (EI, BCI, Callosal Angle) pre- and post-ELD were not significant. However, the differences between the z-Evans index, a 3-Directional linear measure, pre- and post-ELD was significant within the NPH cohort.*

When we classified patients by their testing timepoints, morphological indices alone were insufficient to distinguish between Responders vs. Non-Responders to CSF drainage, at either pre-or post-ELD (Table 6). However, when we classified patients by their responsiveness to ELD, changes in 3d morphological indices across timepoints were able to distinguish between Responders vs. Non-Responders to lumbar testing. The effect of ELD resulted in a significant reduction of 3d morphological indices; this only occurred in Non-Responders and was consistent across multiple independently derived measures (Table 7). These effects include a decrease in z-Evans Index (*p* = 0.001) and an increase in BVR at the level of the PC (*p* = 0.024), due to a decrease in the ventricular component of the BVR (*p* = 0.005). 3D volumetric analysis also supported the significant decrease in ventricular volumes, only in Non-Responders post-drainage (*p* = 0.008).

**TABLE 6:** Classification of normal pressure hydrocephalus (NPH) cohorts by timepoints pre- vs. post-lumbar testing: morphological indices within groups compared by their responsiveness to external lumbar drainage (ELD).

	Pre-ELD			Post-ELD		
Measurements	Responders (*n* = 9)	Non-responders (*n* = 12)	p-value	Responders (*n* = 9)	Non-responders (*n* = 12)	*p*-value
**Traditional 2d linear measures**						

EI	0.402 ± 0.054	0.363 ± 0.048	0.100	0.399 ± 0.045	0.360 ± 0.050	0.079
BCI	0.291 ± 0.026	0.278 ± 0.027	0.298	0.294 ± 0.028	0.274 ± 0.031	0.127
Callosal angle (degrees)	61.6 ± 21.0	53.8 ± 21.0	0.413	61.3 ± 19.7	55.8 ± 21.1	0.548

**Novel 3-directional linear measures**						

z-Evans index	0.461 ± 0.061	0.438 ± 0.052	0.359	0.460 ± 0.059	0.424 ± 0.049	0.152
BVR at AC	0.613 ± 0.172	0.726 ± 0.170	0.151	0.616 ± 0.159	0.714 ± 0.216	0.263
BVR at PC	0.789 ± 0.361	0.869 ± 0.290	0.581	0.773 ± 0.295	0.926 ± 0.269	0.230

*ELD, External lumbar drain.*

*EI, Evans index.*

*BCI, Bicaudate index.*

*BVR, Brain-ventricle ratio.*

*AC, Anterior commissure.*

*PC, Posterior commissure.*

*Comparison of morphological indices between NPH response groups at pre- and post-ELD timepoints. We first classified patients by their drainage timepoints. Within the cohort, morphological indices were insufficient in distinguishing between Responders to Non-Responders of ELD at either timepoints.*

**TABLE 7:** Classification of normal pressure hydrocephalus (NPH) cohorts by Responsiveness to CSF drainage: Morphological indices within groups compared at baseline vs. post-lumbar testing.

	Responders (*n* = 9)			Non-responders (*n* = 12)		
Measurements	Pre−ELD	Post−ELD	*p*-value	Pre−ELD	Post−ELD	*p*-value
**Traditional 2d linear measures**						

EI	0.402 ± 0.054	0.399 ± 0.045	0.789	0.363 ± 0.048	0.360 ± 0.050	0.091
BCI	0.291 ± 0.026	0.294 ± 0.028	0.357	0.278 ± 0.027	0.274 ± 0.031	0.215
Callosal angle (degrees)	61.6 ± 21.0	61.3 ± 19.7	0.866	53.8 ± 21.0	55.8 ± 21.1	0.201

**Novel 3-directional linear measures**						

z-Evans index	0.461 ± 0.061	0.460 ± 0.059	0.811	0.438 ± 0.052	0.424 ± 0.049	0.001*
BVR at AC	0.613 ± 0.172	0.616 ± 0.159	0.833	0.726 ± 0.170	0.714 ± 0.216	0.757
BVR at PC	0.789 ± 0.361	0.773 ± 0.295	0.643	0.869 ± 0.290	0.926 ± 0.269	0.024*

**BVR at PC**						

Brain	27.36 ± 5.583	27.12 ± 5.392	0.589	29.58 ± 4.774	30.23 ± 4.335	0.234
Ventricle	38.60 ± 0.490	38.22 ± 9.639	0.502	35.68 ± 5.772	33.89 ± 5.260	0.005*

**3-Dimensional Volumetric Analysis**						

Ventricular volume (cm^3^)	160.56 ± 89.9	120.28 ± 39.0	0.388	156.05 ± 77.5	114.16 ± 38.0	0.008*

*Significant at α < 0.05.

*ELD, External lumbar drain.*

*EI, Evans index.BCI, Bicaudate index.*

*BVR, Brain-ventricle ratio.*

*AC, Anterior commissure.*

*PC, Posterior commissure.*

*Comparison of morphological indices between drainage timepoints within NPH response groups. We further classified patients by their responsiveness to ELD. Within these subgroups, changes in 3-directional morphological indices (z-Evans index and BVR at PC) across timepoints were able to distinguish between Responders vs. Non-Responders to lumbar testing. The effect of ELD resultsed in a significant reduction of morphological indices specifically in Non-Responders. This finding was consistent across multiple indipendetly derived measures (Ventricle component of BVR at PC, and 3-Dimentional volumentric analysis).*

## Discussion

In this study, we examined the impact of multimorbidity burden, frailty risks and the efficacy of novel 3d linear measures in the Asian context of hydrocephalus vs. non-hydrocephalus and Classic vs. Complex NPH subtypes. Comorbidities, such as hypertension (81%), diabetes mellitus (33.3%) and myocardial infarction/percutaneous coronary intervention/angina (3%), were commonly found in our subjects. A higher comorbidity profile is known to decrease the chances of favorable outcomes in NPH patients who undergo ventricular shunting (Meier and Lemcke, 2008). After ELD, we found that there was a higher proportion of Non-Responders as compared to Responders in our population. Given that ELD is known to accurately predict responsiveness to long-term ventricular shunting (Walchenbach et al., 2002), this suggests that a majority of our study population would not benefit from definitive ventricular shunting procedures (Chotai et al., 2014; Gallina et al., 2018). These data are consistent with expectations of reversibility of the NPH component in patients presenting with multiple comorbidities. Conversely, our study also provided evidence for the fallacies of screening NPH patient cohorts using comorbidities and frailty risks alone. Neither of these risks, nor traditional 2d linear radiological measures, were sufficient to predict CSF responsiveness within our cohort of Classic and Complex NPH subtypes.

To investigate the frailty risks of our population, we stratified our patients by means of a well-validated frailty score, in order to quantify biological, rather than purely chronological, age (Farhat et al., 2012; Pazniokas et al., 2020). Using mFI-11, we categorized our patient population into two discrete groups and assessed if there was any correlation with responsiveness to ELD. We found that a higher frailty score was neither correlated with CSF responsiveness nor the increased likelihood of being termed to have Complex NPH according to our criteria. Whilst there have been no definitive studies studying the link between frailty and shunt responsiveness in NPH patients, a recent study found that frailty was not associated with early post-operative patient-related complications post-ventricular shunting (Hadjiathanasiou et al., 2020). However, we should note that the study did not include patients with high frailty scores in their cohort, similar to our study in which the highest mFI-11 score was 6, out of a maximum of 11. Inclusion of patients with higher frailty scores may have led to different findings and should be the focus of future studies. Frailty has also been reported to be correlated with CNS elastance coefficient, suggesting that frailty is associated with pathological brain aging and the development of neurodegenerative diseases (Vallet et al., 2020). We would then expect that even if frailty was unable to predict CSF responsiveness, it would be significantly correlated to our Complex NPH group in which the proportion of neurodegenerative diseases are higher. However, our results suggest that frailty risks, which are also influenced by the presence of comorbidities, are likely to occur as an irreversible entity separate to the presentation of patients having what we term to be the subtype of Complex NPH. Instead, in Complex NPH, we believe the distinction to be that the probable/possible reversible NPH component still co-exists, except it does so in the presence of challenging overlay from irreversible risks of vascular risk burden/neurodegenerative disorders (that also contribute to the frailty score). Population differences (Asian vs. Caucasian) and our relatively small sample size may account for some of these discrepancies and global studies focused on patients with Complex NPH would be required to draw meaningful conclusions.

The association between changes in ventricular morphology and volumes with respect to ELD-responsiveness has been reported. Both Anderson et al. (2002), and more recently, Neikter et al. (2020), have shown that NPH responders demonstrate a significant decrease in ventricular volumes post-shunting. Yamada et al. (2019) also described increases in BVRs and reductions in ventricular volumes being correlated with excellent outcomes in NPH patients. In patients with idiopathic NPH, it has been shown that bilateral ventricle expansions occur in the z-axial direction, as opposed to the x-axial direction (Neikter et al., 2020). Thus 3d linear measurements, such as the z-Evans Index and BVRs, may be a better approximation of actual changes in brain-ventricular volumes as compared to traditional 2d indices (Neikter et al., 2020).

Interestingly, when we stratified our data according to responsiveness to ELD, this change in ventricular morphology was only significant amongst the Non-Responders. Changes in BVR values matched with a decrease in ventricular volumes, as demonstrated by Yamada et al. (2019). However, whilst these findings appear contradictory, we believe the changes to be reflective of the composition of our NPH cohort, with its large proportion of the Complex NPH subtype. In Complex NPH, the concurrent load of vascular risk burden and/or neurodegenerative diseases may contribute to brain changes more consistent with irreversible injury, such as atrophy and loss of tissue microstructure or elasticity. Conversely, such changes may promote the ease with which fluid moves between CSF compartments during test challenges such as ELD. As 3d linear measures are sensitive to changes in both ventricular volume and apparent overlying brain thickness due to distortion, large passive fluid movements would also be described by these morphological indices. Indeed, our negative results in Responders are also consistent with findings by Meier and Mutze (2004), and Lenfeldt et al. (2012), who demonstrated clinical improvement in NPH patients post-ELD without significant alterations in ventricular size and volume. As a minimum, our data have shown that significant reduction in intracranial CSF compartments was achieved *via* ELD in the Non-responder group to confirm that their lack of responsiveness was not confounded by insufficient CSF drainage during testing. However, taken together with findings from literature, our study also suggests that, using changes in 3d linear measures, it may also be possible to describe the spectrum of brain-ventricular changes across both Classic and Complex NPH subtypes. Significant changes in BVR and ventricular volumes may be present at both extremes of reversibility and irreversibility of brain injury patterns in NPH, reflecting differing underlying pathophysiological processes, from brain compression/distortion to alterations in its poroelastic properties.

There is evidence from other modalities of imaging to support the impact of changes in brain microstructure on CSF movement between intracranial fluid compartments. Brain compression and stiffness in nonlinear elastic regions, as seen on Magnetic Resonance Elastography (MRE) have been hypothesized to lead to non-compliance, which may result in increased CSF drainage from the lateral ventricles, with no change in clinical response (Fattahi et al., 2016). Bugalho and Alves (2007) described irreversibility of NPH symptoms in patients with higher loads of white matter lesions on MRI analysis, suggesting that such parenchymal white matter lesions, thought to be a hallmark of brain microvascular disease, may result in poor response to ventricular shunting. Differential regions of stiffness on MRE imaging, in the temporal lobe for instance, may also portend failure in response to ventricular shunting (Perry et al., 2017). Our study cohort comprises predominantly Complex NPH patients, in which higher levels of brain atrophy and neurodegeneration would be expected as compared to cohorts of purely Classic NPH. Here, differences in ventricular morphology could be explained by loss of microstructural integrity, resulting in both the larger increase in BVR at PC in Non-Responders and the reduction in ventricular volumes. As the BVR measure describes a ratio, a significant drop of ventricular volume (the denominator) in our cohort would drive the larger increase in BVR and be reflected in the z-Evans Index and 3D volumetric analysis as a significant reduction in ventricular size and volume. Conversely, the same changes would still be consistent with that reported by Yamada et al. (2019) in his purer cohort of Classic NPH. In this context, a significant rise of the brain measurement (the numerator), due to improvement in compression, would also drive a larger increase in BVR and be reflected in the z-Evans Index and 3D volumetric analysis as a significant reduction in ventricular size and volume, due to successful CSF drainage *via* ELD. However, the differences between our two cohorts across the spectrum of NPH would be that, for the same significant volume of changes in ventricular morphology, loss of microstructural integrity would likely be less reversible than pathophysiological processes such as brain compression, stretching or tissue distortion.

In further unpacking such considerations, Diffusion Tensor Imaging (DTI), a methodology of modeling changes in white matter microarchitectural patterns by the use of water diffusion properties, may be a helpful adjunct. We have previously described the utility of DTI to describe differing concurrent changes in white matter injuries in response to NPH and interventions, across Classic (Keong et al., 2017) and Complex subtypes (Lock et al., 2019). Indeed, in our DTI analysis of the effect of ELD on Complex NPH patients, we described that a global reduction of DTI metrics across all measures was consistent with passive fluid movement across compartments, suggestive of axonal disruption and brain atrophy. In addition, DTI profiles of white matter injury patterns in Responders still also demonstrated evidence of axonal disruption. However, despite the multiple changes occurring concurrently, we found that the DTI tissue signature of predominant stretch/compression, i.e., the white matter injury pattern most amenable to surgical intervention, was still consistent across Classic and Complex NPH cohorts (Lock et al., 2019). Such white matter changes are compatible with hypotheses suggested by the studies above involving 3d linear measures. Further work is needed to explore the coupling of brain-ventricular concepts in hydrocephalus and neurodegenerative cohorts *via* the concurrent use of 3d, as well as 3D, structural morphological indices and DTI methodology.

### Limitations

Limitations of this study includes the relatively small sample size of our cohort, restricting the statistical power of the study. Nonetheless, testing of morphological indices on this small disease cohort yielded significant findings, consistent with other published work, which are encouraging for future studies to be conducted on larger NPH cohorts with multimorbidity burden and overlay from neurodegenerative disorders. Secondly, our methods of ventricular segmentation were semi-automated *via* 3D Slicer with manual exclusion of falsely included CSF-intensity voxels following anatomic identification by the software. This could have led to human error and inter-operator variability. However, this approach is consistent with similar methods performed *via* the radiology workstation. We minimized these considerations by having a standard operating protocol, refined by pilot testing and incorporating all subsequent post-processing steps within a continuous workflow for consistency, using available modules on 3D Slicer. Lastly, our patient population comprised a large proportion of patients with Complex NPH, and whilst this is reflective of the clinical practice in the Asian population, the impact of other concomitant neurodegenerative diseases may have influenced our results, as compared to the purer cohorts of Classic NPH previously published on the use of morphological indices.

## Conclusion

Our study has shown that novel 3-directional (3d) linear indices can be applied to cohorts of Classic and Complex NPH, and to distinguish them from AD and HC cohorts. We also demonstrated that, contrary to traditional 2d linear measurements, these measures can also provide significant correlations to CSF responsiveness and 3D ventricular volumetry. In the context of multimorbidity burden and overlay from neurodegenerative disease, 3d morphological indices may provide a non-invasive tool to aid in the characterization of NPH cohorts at the clinical-research interface.

## Data Availability Statement

The datasets presented in this article are not readily available because de-identified data is not allowed to be made available to the public according to the study’s IRB. Requests to access the datasets should be directed to NK, nk330@cantab.net.

## Ethics Statement

The studies involving human participants were reviewed and approved by the SingHealth Centralised Institutional Review Board (CIRB; Ref 2016/2627). The patients/participants provided their written informed consent to participate in this study.

## Author Contributions

NK was primarily involved in the study design, protocol development, protocol implementation and analysis of the data at study site, as well as patient recruitment. SS, CL, SK, and JK were involved in patient recruitment and data collection. AT was involved in the frailty scoring. YL was involved in the 3D volumetric analysis. SS and AK were involved in the manuscript preparation with NK. CL and SS aided with the data analysis and statistical prowess. All authors have read and approved the final manuscript.

## Conflict of Interest

The authors declare that the research was conducted in the absence of any commercial or financial relationships that could be construed as a potential conflict of interest.

## Publisher’s Note

All claims expressed in this article are solely those of the authors and do not necessarily represent those of their affiliated organizations, or those of the publisher, the editors and the reviewers. Any product that may be evaluated in this article, or claim that may be made by its manufacturer, is not guaranteed or endorsed by the publisher.

## Footnotes

1
adni.loni.usc.edu/study-design/

2
adni.loni.usc.edu/methods/documents/mri-protocols/

3
www.slicer.org

4
www.r-project.org/foundation

## Supplementary Material

Supplementary Figure 1 Validation of 3D Slicer workflow. NPH, Normal Pressure Hydrocephalus; AD, Alzheimer’s Disease; HC, Healthy Control.
