# Supplementary figures and images for: The Impact of Multimorbidity Burden, Frailty Risk Scoring, and 3-Directional Morphological Indices vs. Testing for CSF Responsiveness in Normal Pressure Hydrocephalus
Source: Front Neurosci. 2021 Nov 16;15:751145. doi: 10.3389/fnins.2021.751145 (PMC8636813; doi:10.3389/fnins.2021.751145)

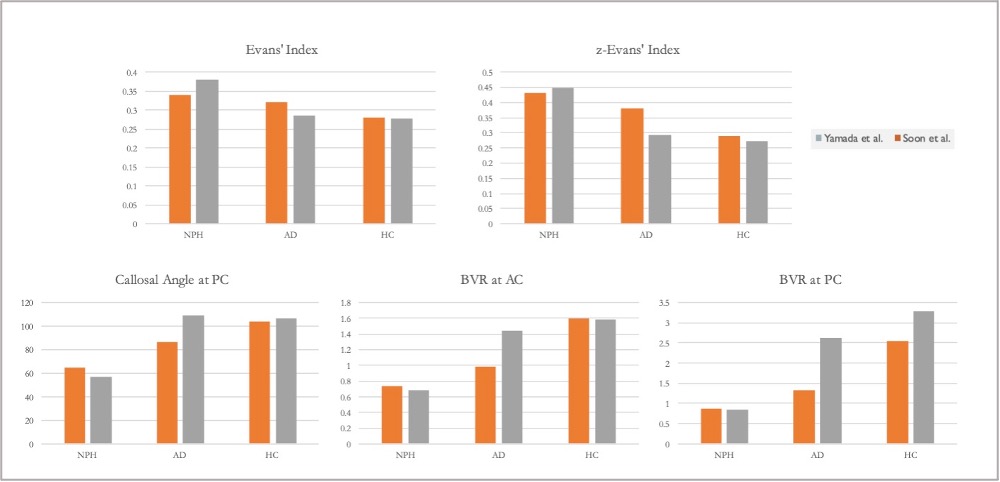

Supplement: Supplementary Figure 1 — Validation of 3D Slicer workflow. NPH, Normal Pressure Hydrocephalus; AD, Alzheimer’s Disease; HC, Healthy Control. [file Image_1.JPEG]
